# *N*-Myristoyltransferase, a Potential Antifungal Candidate Drug-Target for Aspergillus flavus

**DOI:** 10.1128/spectrum.04212-22

**Published:** 2022-12-21

**Authors:** Yu Wang, Ranxun Lin, Mengxin Liu, Sen Wang, Hongyu Chen, Wanlin Zeng, Xinyi Nie, Shihua Wang

**Affiliations:** a State Key Laboratory of Ecological Pest Control for Fujian and Taiwan Crops, Fujian Agriculture and Forestry University, Fuzhou, Fujian, China; b Key Laboratory of Pathogenic Fungi and Mycotoxins of Fujian Province, Fujian Agriculture and Forestry University, Fuzhou, Fujian, China; c School of Life Sciences, Fujian Agriculture and Forestry University, Fuzhou, Fujian, China; National University of Singapore and Genome Institute of Singapore

**Keywords:** *N*-myristoyltransferase, *Aspergillus flavus*, crystal structure, spore, sclerotia, aflatoxin, protein prediction, AlphaFold2

## Abstract

The filamentous fungus Aspergillus flavus causes devastating diseases not only to cash crops but also to humans by secreting a series of secondary metabolites called aflatoxins. In the cotranslational or posttranslational process, *N*-myristoyltransferase (Nmt) is a crucial enzyme that catalyzes the myristate group from myristoyl-coenzyme A (myristoyl-CoA) to the N terminus or internal glycine residue of a protein by forming a covalent bond. Members of the Nmt family execute a diverse range of biological functions across a broad range of fungi. However, the underlying mechanism of AflNmt action in the A. flavus life cycle is unclear, particularly during the growth, development, and secondary metabolic synthesis stages. In the present study, *AlfNmt* was found to be essential for the development of spore and sclerotia, based on the regulation of the xylose-inducible promoter. AflNmt, located in the cytoplasm of A. flavus, is also involved in modulating aflatoxin (AFB1) in A. flavus, which has not previously been reported in Aspergillus spp. In addition, we purified, characterized, and crystallized the recombinant AflNmt protein (rAflNmt) from the Escherichia coli expression system. Interestingly, the crystal structure of rAlfNmt is moderately different from the models predicted by AlphaFold2 in the N-terminal region, indicating the limitations of machine-learning prediction. In conclusion, these results provide a molecular basis for the functional role of AflNmt in A. flavus and structural insights concerning protein prediction.

**IMPORTANCE** As an opportunistic pathogen, A. flavus causes crop loss due to fungal growth and mycotoxin contamination. Investigating the role of virulence factors during infection and searching for novel drug targets have been popular scientific topics in the field of fungal control. Nmt has become a potential target in some organisms. However, whether Nmt is involved in the developmental stages of A. flavus and aflatoxin synthesis, and whether AlfNmt is an ideal target for structure-based drug design, remains unclear. This study systematically explored and identified the role of AlfNmt in the development of spore and sclerotia, especially in aflatoxin biosynthesis. Moreover, although there is not much difference between the AflNmt model predicted using the AlphaFold2 technique and the structure determined using the X-ray method, current AI prediction models may not be suitable for structure-based drug development. There is still room for further improvements in protein prediction.

## INTRODUCTION

Aspergillus flavus is one of the most ubiquitous plant-pathogenic fungi worldwide. First receiving scientific attention in the 1960s (1), this notorious aflatoxin-producing species is responsible for serious economic losses in crop production during pre-harvest, harvest, and post-harvest (1). Aflatoxins are a series of secondary metabolites synthesized by A. flavus and other Aspergillus *species* (e.g., A. parasiticus). Among these toxins, AFB1 is the most abundant and most toxic. Long-term consumption of aflatoxin-contaminated food has carcinogenic effects on humans and animals. In addition, as a human opportunistic pathogen, A. flavus is a major causal agent of fungal infections (2). More than 200,000 people per year around the world are diagnosed with invasive aspergillosis due to Aspergillus infections (3). Patients with respiratory diseases such as COVID-19 are susceptible to these invading fungal pathogens, which may aggravate illness (4).

Nowadays, commonly fungal inhibitors are roughly divided into three categories based on their compound chemical structure: polyenes (amphotericin B) (5, 6), azoles (voriconazole) (7), and echinocandins (caspofungin) (8). In recent years, two novel agents, Olorofim (formerly F901318) and Brexafemme (formerly SCY-078), have undergone clinical development evaluation for the treatment of fungal infections (9, 10). In the fields of fungal control and plant protection, studying the role of virulence factors during the infection process and the search for novel drug targets have been hot scientific topics. Cell wall synthesis-related proteins (β-1,3-glucan synthase) (8), lipid-anchored proteins (glycosylphosphatidylinositol-linked protein) (11), and enzymes (14-demethylases) in the sterol biosynthesis pathway are groups of characterized drug targets with crucial physiological significance (12).

*N*-myristoyltransferase (Nmt), which belongs to the GCN5 acetyltransferase superfamily, catalyzes the myriastate group from myristoyl-coenzyme A (myristoyl-CoA) to the N terminus or internal glycine residue of a protein by forming a covalent bond (13). This catalytic modification, reaction known as *N*-myristoylation, occurs in organism cotranslational or posttranslational processing (14). With the application of improved mass spectrometry proteomics technology, more than 100 *N*-myristoylation substrate proteins have been identified (15), such as tyrosine protein kinase Src, ADP ribosylation factor, and tyrosine protein kinase Fyn (15). In filamentous fungi, *N*-myristoylation is associated with ubiquitin-mediated degradation during cellular morphogenesis (16). Typically, humankind has two Nmt isozymes, while there is only one Nmt coding-gene copy in fungi (e.g., Saccharomyces cerevisiae, Candida albicans, and A. fumigatus) (17–21). Nmt is an essential enzyme for the viability of clinically relevant pathogenic fungi such as C. albicans and Cryptococcus neoformans (19, 20). An Nmt mutant strain of C. albicans lost the ability to infect the mouse respiratory system (22). In A. fumigatus, Nmt is necessary for cell wall morphogenesis and cell polarity establishment (21). Moreover, there is some evidence that supplementation of myristate can partially restore the Nmt-deficient cell phenotype in S. cerevisiae (17), C. neoformans, and A. nidulans (17, 23, 24). Nevertheless, the biological function of AflNmt in A. flavus is unclear, and whether it is an appropriate drug target for the prevention and control of the fungus remains to be verified.

Here, we report that AlfNmt, which is predominantly localized in the cytoplasm, has meaningful effects on the organismal phenotypes of A. flavus, including colony formation, mycelial germination and growth, sporulation, sclerotium production, and aflatoxin biosynthesis. In addition, we purified and crystallized the recombinant NMT protein (rAfNmt) from the prokaryotic expression system, and the experimental crystal structure is slightly different from AlphaFold2 predicted models in the N-terminal region of protein. These structural conformational comparisons may help minimize bias in the AI learning process and result in better performance for computational model building. These findings provide comprehensive information for understanding the regulation of AlfNmt in A. flavus and the development of fungistatic agents in the near future.

## RESULTS

### *AflNmt* is a lethal single-copy gene.

**FIG 1:**
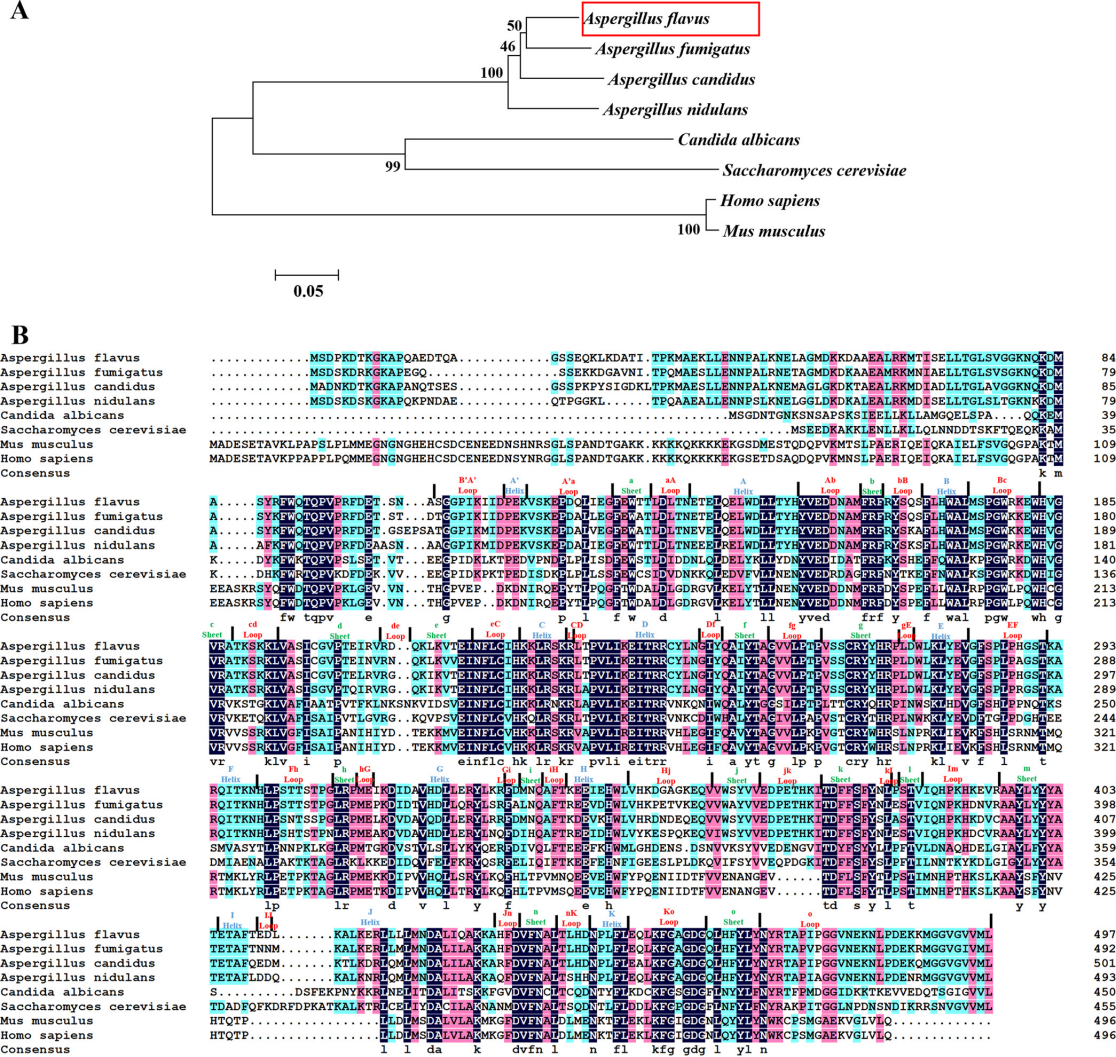
Identification of an Nmt-encoding gene in Aspergillusflavus. (A) A phylogenetic tree of Nmt homologous proteins (*A. flavus*, *A. fumigatus*, *A. candidus*, *A. nidulans*, *Candida albicans*, *Saccharomyces cerevisae*, *Mus musculus*, and *Homo sapiens*) was constructed by MAGA program (version 5.0). (B) Sequence alignment illustration of the Nmt proteins was generated using ESPript (https://espript.ibcp.fr/ESPript/cgi-bin/ESPript.cgi). Strictly conserved residues are highlighted in differently colored boxes, and secondary structural elements from the crystal structure of rAlfNmt are shown above the sequences.

The Nmt amino acid sequences for A. flavus, A. fumigatus, A. nidulans, C. albicans, Homo sapiens, and Mus musculus were analyzed by the phylogenetic tree method (Fig. 1A). As shown in Fig. 1A, the amino acid sequence of AflNmt was similar to those of filamentous fungi, especially Aspergillus species, but it was considerably different from other orthologs (such as H. sapiens). As determined by multiple sequence alignment analysis (Fig. 1B), the substrate recognition region is moderately distinct between AflNmt and HsNmt; for example, in the m β-sheet region.

We first attempted to construct a deletion strain (Δ*AlfNmt*) using homologous recombination as described previously (25), but not to generate positive transformants in screening medium. A variety of fungi carry a single copy of *Nmt* in their own genomes, and these *Nmt* genes are essential in most cases (19, 21). In view of that, we confirmed that A. flavus contained only a single copy of *AflNmt*, the same as that of the *AflSumo* reference gene, by using quantitative real-time PCR (Table S4) (25, 26). Alternately, we used a conditional gene expression strategy employing a xylose-inducible promoter to investigate *Nmt* function in A. flavus (Fig. S1A) (27). A fusion PCR product with nutritional marker (*pyrG*), xylose-inducible promoter, and *Nmt* was transformed into A. flavus CA14 PTS strain to generate a xylose control strain (*AflNmt^xylP^*; Fig. S1B), then the transformed positive cells were confirmed via PCR assay (Fig. S1C). To further validate the construction of the *AflNmt^xylP^* mutant, the transcriptional expression of *AflNmt* was analyzed using reverse transcription quantitative PCR. As shown in Fig. S1D, the expression level of *AflNmt* in the *AflNmt^xylP^* strain induced by xylose was extremely low compared with that in the wild-type (WT) strain, confirming successful promoter replacement in the mutant strain.

### AflNmt is necessary for colony growth, spore germination, and sclerotia formation in *A. flavus*.

**FIG 2:**
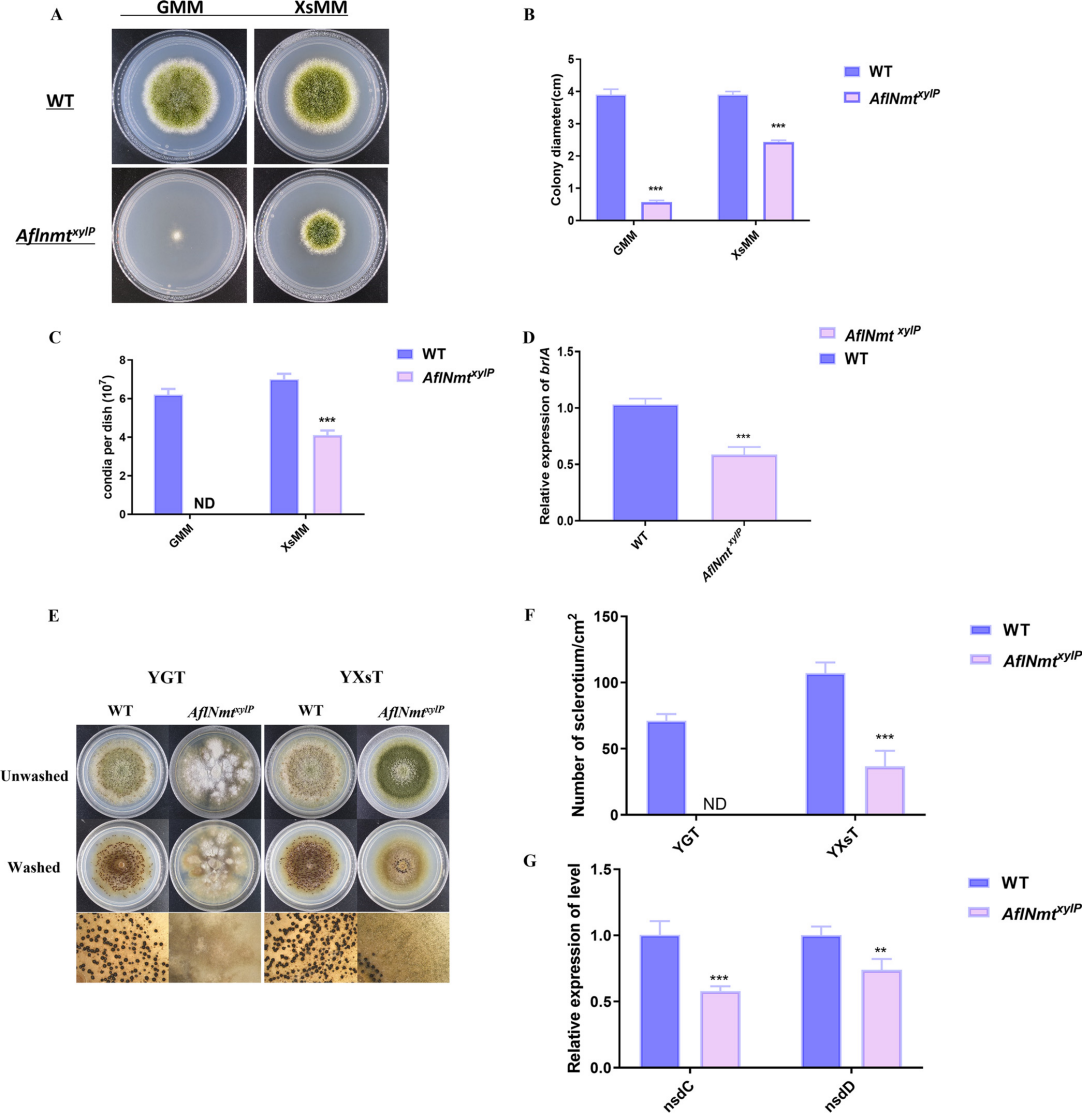
AflNmt regulates colony and conidia development and sclerotia formation in A. flavus. (A) Phenotypic observations of the wild-type (WT) and *AflNmt^xylP^* strains in Glucose Minimal Medium (GMM) or Xylose Minimal Medium (XsMM) solid medium for 18 h at 37°C, respectively. (B) Colony diameters were compared in GMM and XsMM medium for these two A. flavus strains. (C) Numbers of conidia produced by the two strains. (D) Relative transcript expression levels of the conidia marker gene *brlA* in XsMM solid medium for the two strains. (E) Microscopic view of sclerotium formation of WT and *AflNmt^xylP^* strains in yeast-glucose-trace element (YGT) or yeast-xylose-trace element (YXsT) solid medium. (F) Amount of sclerotia produced by the different A. flavus strains. (G) Relative transcript expression levels of the sclerotia biosynthesis genes *nsdC* and *nsdD* in XsMM solid medium for the two strains. ***, *P* < 0.001; **, *P* < 0.01 based on *t* tests with three biological replicates.

One of the virulence factors in filamentous fungi is colonization of the host by hyphal extension. To investigate the effects of AflNmt in the colony growth of A. flavus, two strains (wild-type and *Nmt^xylP^*) were cultured in Glucose Minimal Medium (GMM) and Xylose minimal medium (XsMM), respectively. The growth of the *AflNmt^xylP^* strain was significantly inhibited compared with that of the WT strain in GMM medium, and visual observations showed that aerial mycelia were scarce in the center of the inoculated plate for the mutant strain (Fig. 2A). The defective phenotype of *AflNmt^xylP^* was moderately restored upon xylose supplementation (Fig. 2A). For example, the colony size of *AflNmt^xylP^* recovered to more than three quarters, albeit not as well as that of the wild-type strain (Fig. 2B). Thus, the decline in colony growth and delay in colony size supported the knockdown of *AflNmt*.

Given that conidial germination is an important indicator of propagation in plant-pathogenic fungi, we additionally assessed whether there was a positive correlation between AflNmt and spore-producing capacity in A. flavus. The spore-counting assays demonstrated that the *AflNmt^xylP^* strain lost the ability to produce spores in GMM. The sporulation capacity of *AflNmt^xylP^* was improved by adding xylose molecule, and the spore count of this conditional mutation in XsMM reached 75% that of WT strain (Fig. 2C). Meanwhile, quantitative real-time PCR detected downregulation of the sporulation-related gene *brlA* in the mutant strain, indicating that AflNmt impaired conidia formation via manipulating sporulation-related genes (Fig. 2D). We also observed the influence of *AflNmt* knockdown on hypha tip growth and polarity. The *AflNmt^xylP^* strain exhibited abnormal cell polarity in 16-h liquid cultures, implying defects in germination and tube growth (Fig. S2). These results indicated that AflNmt affects growth, sporulation, and spore germination in A. flavus.

Sclerotia are melanized, coarse-grained pellets formed by mycelia which adapt to gradual changes in a harsh external environment. In this study, after 14 days of incubation in darkness, the *AflNmt^xylP^* strain produced considerably fewer sclerotia than the WT strain in yeast-glucose-trace element (YGT) or yeast-xylose-trace element (YXsT) culture media (Fig. 2E). The sclerotia number for the *AflNmt^xylP^* strain was significantly decreased by roughly 3-fold compared with that of WT strain (Fig. 2F). Two reported transcription factors, *NsdC* and *NsdD*, are considered essential for sclerotia morphology changes in A. flavus. We monitored the expression levels of these two reference genes via quantitative real-time PCR analysis. The transcription levels of *NsdC* and *NsdD* in *AflNmt^xylP^* were dramatically lower than those in the WT strain (Fig. 2G). Molecular and phenotypic identification have thus revealed that AflNmt is a vital factor for sclerotia formation in A. flavus.

### AflNmt is associated with AFB1 production in *A. flavus*.

**FIG 3:**
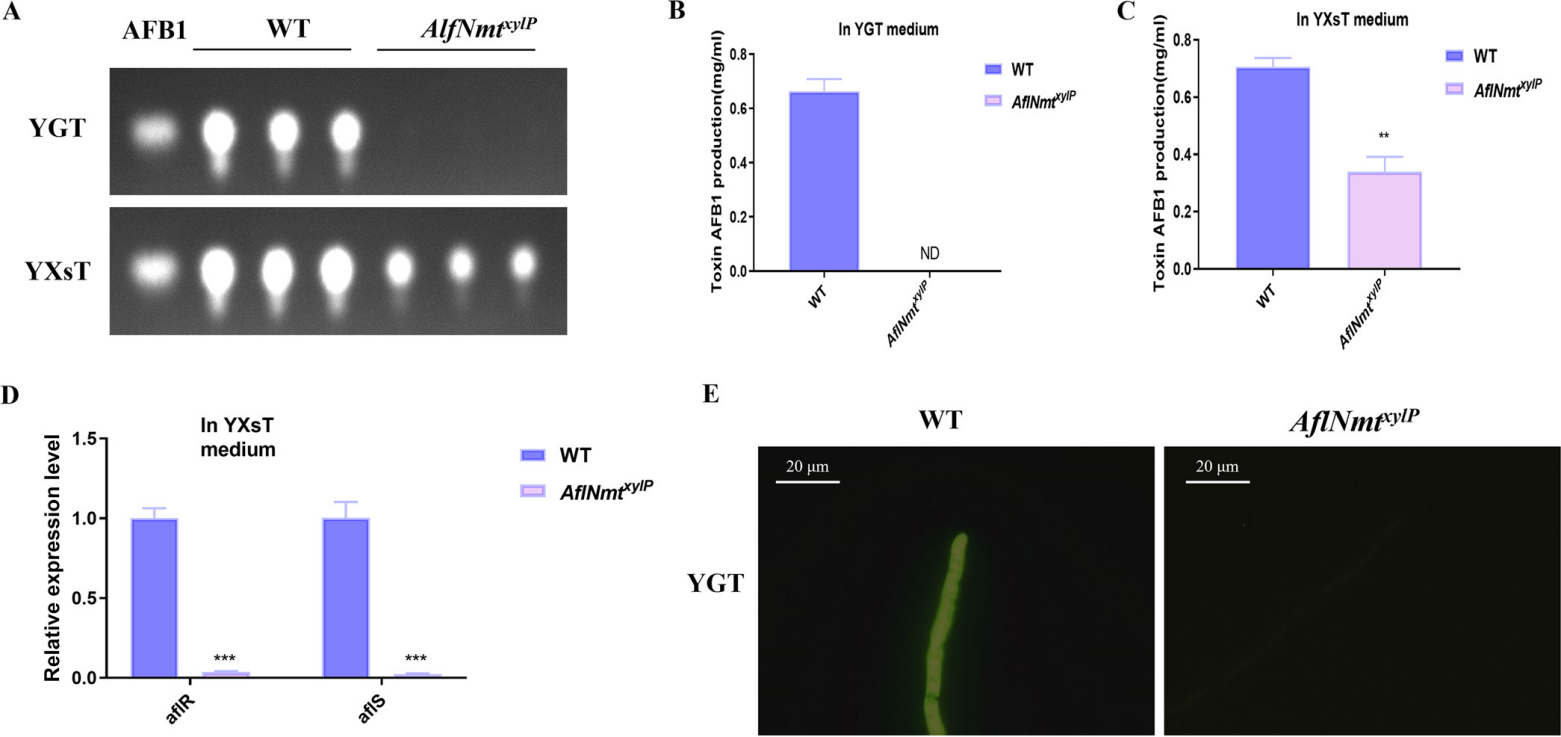
Effects of AflNmt on aflatoxin synthesis. (A) Thin-layer chromatography analysis of AFB1 production for WT and *AflNmt^xylP^* strains with three experimental replicates in YGT or YXsT liquid culture medium. (B, C) Optical density evaluation of AFB1 production (A) in YGT or YXsT liquid culture medium. (D) Relative transcriptional level of aflatoxin synthesis-related genes (*aflR* and *aflS*) in these two strains with three biological replicates in YGT culture medium. (E) Reactive oxygen species production of WT or *AflNmt^xylP^* in YGT culture medium. Scale bar = 20 μm.

The secondary metabolite produced by A. flavus which is of greatest concern is the mutagenic and carcinogenic mycotoxin AFB1. In this study, aflatoxin production was determined by thin-layer chromatography (TLC), which was normalized relative to cultural mycelial biomass. As shown in Fig. 3A to C, the *AflNmt^xylP^* strain was unable to synthesis AFB1 in YGT liquid culture medium and produced only half as much toxin as the WT strain in YXsT medium. In the AFB1 biosynthesis pathway, more than 20 toxin synthesis-associated genes are controlled by two global regulators, *aflR* and *aflS*. The results of real-time PCR suggested that both *aflR* and *aflS* are expressed at drastically low levels in the *AflNmt^xylP^* strain in YGT medium (Fig. 3D). The oxidative stress inside a live fungal cell is positively associated with aflatoxin production (28). Intracellular reactive oxygen species (ROS) signals were observed under a fluorescence microscope by the application of specific dyes (DCFH-DA). Unsurprisingly, the ROS signal of the *AflNmt^xylP^* strain was much weaker than that of the WT strain in YGT liquid culture medium (Fig. 3E). These results indicated that AflNmt may regulate the global regulators in A. flavus to affect aflatoxin metabolism.

### Subcellular localization of AlfNmt.

Nmts derived from other species are most frequently located in the cytoplasm, as in yeasts and humans (29), although Nmt was detected in the human cell membrane in one case (30). To study the subcellular localization of AlfNmt, an *AflNmt*-*GFP* strain was constructed by homologous recombination (Fig. S3A). The blots of *AflNmt*-GFP (green fluorescent protein) were found in the hyphal cytoplasm but did not overlap with those of the fluorescent nuclear dye (DAPI [4′,6-diamidino-2-phenylindole]) under luminescence microscope observation (Fig. S3B), suggesting that AlfNmt exercises its biological functions as a cytoplasmic protein.

### Expression and characterization of recombinant AflNmt.

**FIG 4:**
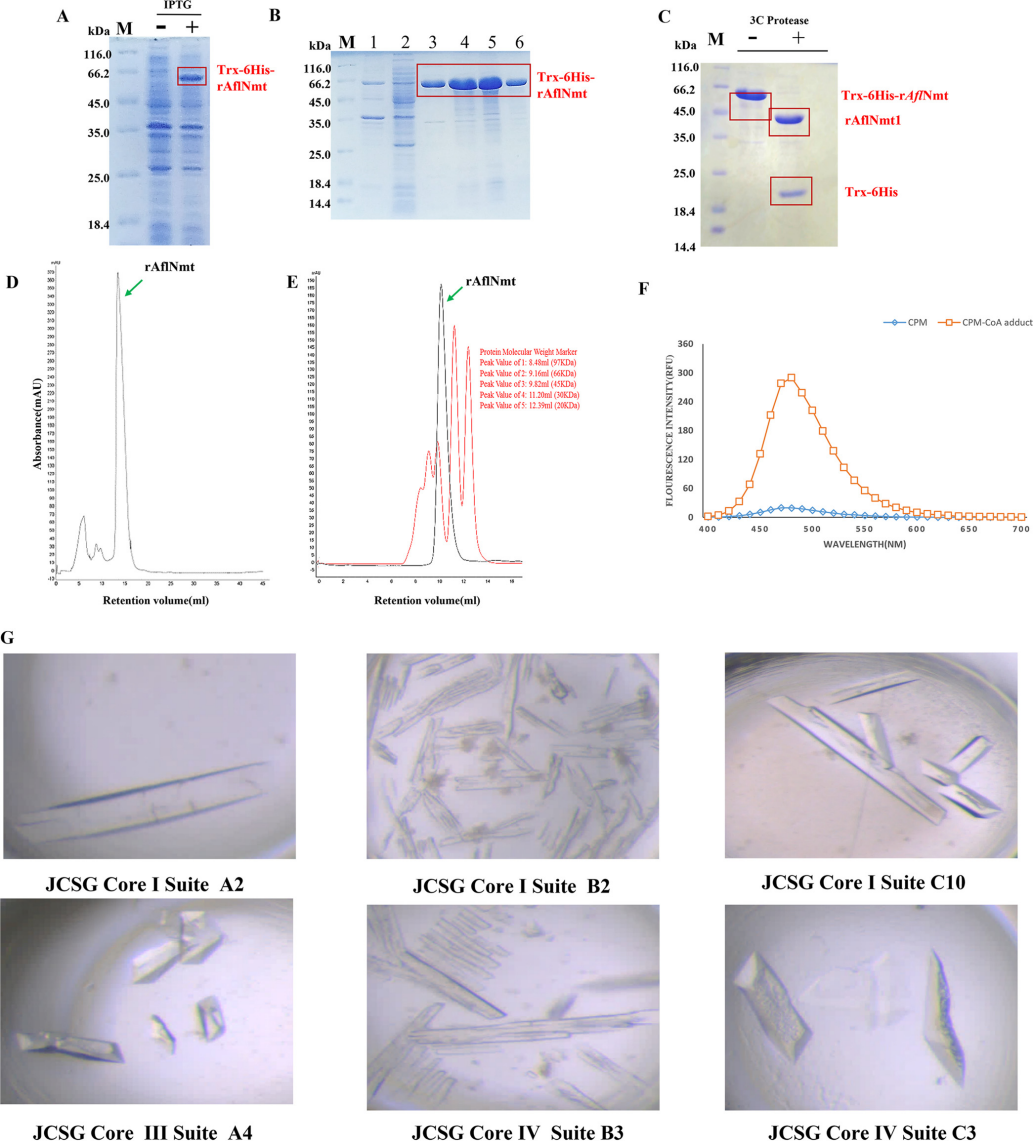
Expression, purification and crystallization of recombinant AflNmt. (A) Target protein expression was induced with 0.3 mM IPTG (isopropyl-β-d-thiogalactopyranoside) for 3 h at 37°C. M: protein marker. (B) Trx-6×His-rAflNmt fusion protein was loaded onto a Ni-NTA column and eluted with a discontinuous gradient concentration (50 mM imidazole and 300 mM imidazole). Lane 1: cell debris. Lane 2: flowthrough fraction. Lanes 3 to 5: proteins were washed and eluted with 50 mM imidazole. Lanes 3 to 6: elution fractions with 300 mM imidazole. (C) Cleavage of the Trex-6×His-tag from fusion protein. (D) Cation exchange chromatography of rAflNmt using a RESOURCE S column. (E) The untagged rAflNMt was purified by molecular sieve chromatography. (F) rAflNmt activity was measured using a nonradioactive fluorescence *in vitro*. (G) A total of 6 conditions yielded rAflNmt protein crystals. JCSG Core I Suite A2 crystallization conditions: 0.1 M bicine (pH 8.5), 20% (wt/vol) PEG 6000 (final pH = 9. JCSG Core I Suite B2 crystallization conditions: 0.1 M HEPES (pH 7.5), 20% (wt/vol) PEG 8000. JCSG Core I Suite C10 crystallization conditions: 0.1 M Tris (pH 7.5), 20% (wt/vol) PEG 8000. JCSG Core III Suite A4 crystallization conditions: 0.1 M CHES (pH 9.5), 30% (wt/vol) PEG 3000. JCSG Core IV Suite B3 crystallization conditions: 0.1 M bicine (pH 8.5), 30% (wt/vol) PEG 6000 (final pH = 9). JCSG Core IV Suite C3 crystallization conditions: 0.17 M sodium acetate, 0.05 Tris-HCl (pH 8.5), 25.5% (wt/vol) PEG 4000, 15% (vol/vol) glycerol.

According to bioinformatic analyses and previously reported protein expression or crystallization strategies, removing the disordered Nmt N terminus in other species facilitates downstream structural study (21, 31, 32). With reference to the homologous protein in *A. fumigatus* (21), the truncated recombinant Nmt protein (Δ1-89 rAflNmt) was expressed as N-terminal Trx-6×His-fusion proteins in an E. coli strain. rAflNmt expression had a relatively low background level and a highly soluble yield in the E. coli expression system (Fig. 4A). The recombinant fusion protein was purified by Ni-column (Fig. 4B). To remove the Trx-6×His-tag, the 3C prescission protease was subsequently employed to cleave the fusion protein. The gel electrophoresis results demonstrated that the digestion of the recombinant fusion protein was efficient (Fig. 4C). The untagged Δ1-89 rAflNmt (43 kDa) and additional fraction (the linker and Trx-6×His-tag peptides) were dialyzed with low-salt solution and then isolated by anion exchange column (RESOURCE S columns; Cytiva, Marlborough, MA) (Fig. 4D). Finally, the target proteins were subjected to Superdex 75 gel filtration chromatography (Fig. 4E). Single peaks of elution fraction were detected from the chromatography profile, indicating that Δ1-89 rAflNmt is consistent with its theoretical molecular weight and exists solely as a monomer in elution buffer. A nonradioactive fluorescent assay was prepared as described in the Methods section to determine protein bioactivity *in vitro*. By observing changes in the fluorescent substrate CPM [7-diethylamino-3-(4-maleimido-phenyl)-4-methylcoumarin], Δ1-89 rAflNmt was shown to possess *N*-myristoyltransferase catalytic activity (Fig. 4F).

### Overall spatial structure of rAflNmt.

The rAflNmt crystal screenings were performed with commercially available screening kits using the sitting drop vapor diffusion method at 16°C. The protein crystals were cultivated under various conditions (Fig. 4G). To attain appropriate diffraction-quality crystals, the crystallization conditions were further optimized.

**FIG 5:**
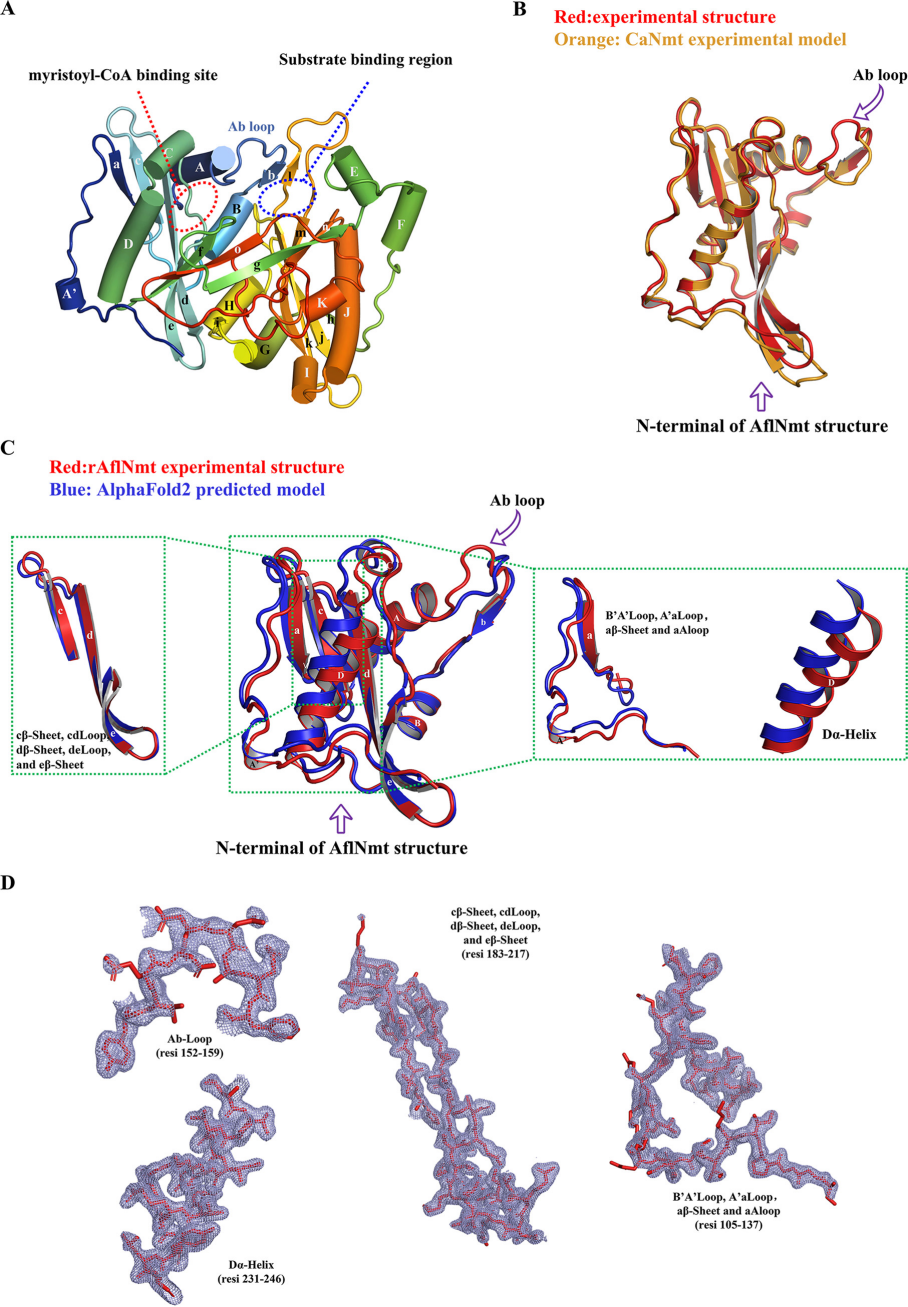
Structural comparison between experimental structure and AlphaFold2-predicted model. (A) Overview of rAflNmt crystal structure. Helix, loop, and β-strand secondary structures for this protein are distinguished by distinct colors. Myristoyl-CoA and substrate binding sites are marked with dotted lines. (B) Structural comparison between experimental structure and AlphaFold2-predicted model in N terminus of AflNmt. The two AflNmt structures are shown with secondary structure elements and colored in red and blue for clarity. (C) Structure comparison of rAflNmt and CaNmt (orange color) at the N-terminal positions. (D) Representative electron cloud density of the *N*-AflNmt structure. The 2Fo-Fc electron density maps were contoured at 1.0 σ and colored in light blue; secondary structure elements are shown as sticks and labeled in red.

The rAflNmt crystal was diffracted to a high resolution of 1.78 Å and was in the P41 space group. The three-dimensional structure of rAflNmt was resolved by the molecular replacement method. The asymmetric unit contained only one rAflNmt molecule, and the structural model was refined to Rwork 0.23 and Rfree 0.26 with Ramachandran (favored 93.6%, allowed 5.1%, and outlier 1.28%). The approximate dimensions of spherical rAflNmt molecule are about 50 × 60 × 55 Å, and this protein is folded into a typical compact α/β topological shape similar to the GCN5-related *N*-acetyltransferase superfamily structure. In the N-terminal or C-terminal subunit, the core of the protein region is formed by several β-sheets and surrounded with a few α-helices (Fig. 5A). In comparison with other Nmt proteins, the myristoyl-CoA binding site and adjacent inhibitor binding pocket were found on the surface of the rAflNmt molecule (Fig. 5A).

### Comparison of AlphaFold2 models and experimental structure for AlfNmt.

AlphaFold2 is a novel machine-learning neural network that predicts tertiary protein structures from the primary sequence with much more precision (33). ColabFold is a rapid and simple online method for implementing the AlphaFold2 algorithm coupled with Google Colaboratory (34). To improve our molecular understanding of the AflNmt protein structure, we generated five homology models of AflNmt using the ColabFold platform. The structure comparison and RMSD (root-mean-square deviation) computation were made with AlphaFold2 models and experimental structure. RMSD is a measure of average distance between the atoms (commonly backbone Cα atoms) for superimposed structures (such as native structure and structures) (35). The RMSD values of the aligned structures in this study were approximately 0.737 to 0.844 Å. To ensure fair evaluation, a prediction model with the minimum RMSD value was further used to compare the AlphaFold2 model against the experimental data. The AlphaFold2-calculated structure agreed very well overall with the experimental X-ray AflNmt structure (Fig. 5C). Major differences between these two structures occurred in the N-terminal region of the enzyme, including the following motifs: Ab loop, αD helix, βc, and βd (Fig. 5C).

## DISCUSSION

### Homologous Nmt exhibited similarities and differences in the regulation of diversified species development.

The Nmt superfamily has been characterized in a wide variety of fungi, such as S. cerevisiae (17), C. neoformans (19), C. albicans (20), Histoplasma capsulatum (36), and A. nidulans (23). Insertional mutagenesis of the Nmt coding gene causes lethal phenotypes in most of the fungal species mentioned above. Although the *Nmt* mutant strain morphology in several species (eg. A. nidulans) can be completely or partially rescued by the addition of myristic acid (23), the *Nmt* deletion experiment in A. fumigatus was unsuccessful with the same supplementation (21). Our experimental data are certainly concordant with those previous studies which also showed that an AflNmt gene knockout strain was not obtained by replacing the target gene with a selective marker (*pyrG*) (21). In addition, the AflNmt coding gene appears to be a single-copy gene associated with essential biological functions in these fungi (21, 23). Accordingly, we speculated that the impact of *Nmt gene* knockout was consistently superior to that of *Nmt* gene knockdown or mutation on fungal cells. As an alternative, we chose a conditional activation strategy that introduces a xylose-inducible promoter, which replaces the native promoter of the *AflNmt* gene.

Polar growth in filamentous fungus is an essential property that contributes to spore, germ tube, and hypha development. In *Nmt*-deficient Aspergillus strains, spore germination is delayed and germ tubes stemming from conidia are kept in a short state over a sustained period of time (21, 23). Our observation of abnormal germination phenotypes is certainly consistent with those of previous studies, indicating that Nmt facilitates the establishment and maintenance of cell polarity in Aspergillus spp.

Although *Nmt* orthologous genes in fungal species showed high sequence conservation, there were slight species-specific differences. With alcohol dehydrogenase promoter, the growth of an *Nmt* mutant strain in media with several inducers (ethanol, glycerol, or threonine) was comparable to that of WT A. fumigatus (21). Nevertheless, in our study, the phenotype of the *AflNmt^xylP^* strain could not be totally recovered by induction with xylose. The phenotypic differences between these two strains may be explained by differences in gene expression, temperature sensitivity, and exogenous additions as follows. In C. neoformans, due to gene-dosage effect, *Nmt* mutation transformants which carry multiple gene copies are prone to grow on potato dextrose agar medium (19). In C. albicans, *Nmt* mutants are sensitive to temperature (such as at 37°C) (19). However, no temperature effect was evident in A. fumigatus at 37 to 50°C or in A. flavus at 37°C (21). Although the addition of myristate into the medium was found to partially restore the growth of *Nmt* mutant cells in S. cerevisiae (24), C. neoformans (19), and A. nidulans (23), this 14-carbon saturated fatty acid is ineffective at rescuing the gene knockout strain in A. fumigatus (21). Furthermore, the downregulation of *AlfNmt* gene expression led to abnormal growth and development, including reduced spore count and impaired sclerotia. The transcription levels of the conidiation regulation gene (*brlA*) and sclerotia-related genes (*nsdC* and *nsdD*) were lower than those in the WT strains, indicating that the biological function of AlfNmt was correlated with several developmental stages of A. flavus.

### AflNmt regulatory role as a key element in the synthesis of aflatoxin.

The secondary metabolic product AFB1 is an essential virulence factor in A. flavus. Interestingly, the defects in *Nmt* impaired aflatoxin biosynthesis in A. flavus, accompanying the low expression of two global regulators of aflatoxin synthesis (*aflR* and *aflS*). To our knowledge, Nmt has never been mentioned as being involved in modulating secondary metabolite production in Aspergillus species. A relatively high degree of sequence conservation for Nmt protein exists in A. flavus, A. nidulans, and A. fumigatus (Fig. 1A and B). A. nidulans generates an important carcinogenic mycotoxin known as sterigmatocystin (37), an intermediate material also produced by A. flavus and A. parasiticus. However, the mycotoxin metabolite fumagillin, biosynthetic gene clusters, and reaction pathways in A. fumigatus are somewhat different from those of the other two pathogenic fungi (38). To find potential myristoylation sites in aflatoxin synthesis-associated proteins, we used two myristoylation site predictors (https://mendel.imp.ac.at/myristate/SUPLpredictor.htm and https://web.expasy.org/myristoylator/). An online search revealed that neither myristoylation site matched AflR or AflS. Therefore, the regulation role of AflNmt-mediated protein myristoylation in the progress of aflatoxin synthesis needs to be further explored.

### AflNmt appears to be a potential antifungal target for the prevention and control of mold infection.

In fact, the Nmt family is widely present in fungi, plants, and animals. Two isoforms of Nmt which share about 77% peptide sequence identity exist in mammalian species, including humans and mice, and are referred to as Nmt1 and Nmt2 (39). Nmt1 has been considered a promising therapeutic target for its high expression in numerous cancer cells (40–42). For lower eukaryotes, Nmts have also become targets for antifungal and anti-parasitic drug development (43, 44). AflNmt has been selected as a novel antifungal target for the prevention and control of mold infection for the following reasons. (i) *AflNmt* gene is essential for survival and proliferation in A. flavus and other pathogenic filamentous fungi. (ii) Unlike the two Nmt isoenzymes in mammals, the A. flavus genome contains only a single Nmt-coding gene, like other fungi (17–21). (iii) Although Nmt is predominantly found in eukaryotes, the recognition of peptide substrate for Nmt is a discrepancy between mammals and fungi. Currently, several Nmt selective inhibitors have been found (36, 45). The bioactivities of Nmts are specifically inhibited by these small-molecule compounds, and the difference in inhibitor selectivity between mammals and fungi can reach 10,000-fold (36, 45). Consequently, these screened or designed drugs could effectively kill infectious pathogens without exerting potentially toxic side effects on the human body.

### Can AlphaFold2 accurately predict the N-terminal structure of AlfNmt.

Recently, DeepMind Technologies Limited has used the AlphaFold2 program to release over 200 million structures for plants, bacteria, animals, and other organisms (46). Prediction of target protein models will help accelerate structural biology research and drug discovery. In both AlphaFold2 models and experimental structures, there are different conformational arrangements in the N terminus of AflNmt. In previous reports, N-terminal amino acids of Nmt have been considered to play a crucial role in ribosomal targeting, substrate myristoyl-CoA recognition, and contribution to peptide catalytic reaction (47–49). The conformation switch in the N-terminal region of Nmt has critical implications for the regulation of enzyme activity. Therefore, we subsequently focused on the conformational changes for these aforementioned motifs. Although a sequence comparison demonstrated that the amino acid residues for the Ab loop (a flexible motif linked with the A helix and β-sheet) were relatively conserved in the Nmt family (Fig. 1B), the flexible Ab loop is present in various conformational isoforms (open, semi-open, and closed) in the protein crystal structure. The structure-function role of Ab loop is controversial for several reasons: (i) the full-length ScNmt structure revealed that the open state of the Ab loop may be related to myristoyl-CoA substrate recognition but not to peptide substrate binding or catalytic action (31), and (ii) in other studies, the flexible Ab loop seemed to induce substrate myristoyl-CoA transient stabilization in the Nmt catalysis process, which has been confirmed by biochemical and structural evidence (50).

In our crystal structure, the Ab loop of AflNmt was in the open conformation, which is different from the structures provided by AlphaFold2 programmer (Fig. 5C). AlphaFold2 introduces a statistical value called pLDDT to evaluate the confidence-per-residue of the predicted structure, in which a higher plDDT score indicates better performance for the model result (51). In the predicted model, the Ab loop score was only between 50 and 70, indicating low confidence (Fig. S1). As a result, it seems that AlphaFold2 does not accurately predict the open or closed state of the Ab loop in the AflNmt structures, as reflected in the pLDDT scores.

Interestingly, the pLDDT for the major N terminus of AlphaFold2 perdition structure was >90, indicating high accuracy and reliability for the given region; however, the predicted model did not align as well with the X-ray structure in the N-terminal zone, and the average carbon backbone RMSD between the two coordinates was 0.924. In addition, we noticed that the N-terminal region of AlfNmt fit adequately into the electron cloud density in the experimental data (Fig. 5D). We speculate that the model bias or inaccurate prediction results are partially based on the deep learning patterns, which are derived from sequence homology and Nmt structures in the PDB database. A database search revealed that the overwhelming majority of N-terminal zones adopted more open conformations in about 100 Nmt family structures, and that these protein models were both binary complex and ternary complexes with myristoyl-CoA, inhibitor, or both. A previous example in the literature of ligand-free Nmt crystal structure supports this possibility: the RMSD of αC between ligand-free CaNMT (PDB: 1NMT) and AflNmt structures is 0.656, which is smaller than the value between the experimental and AlphaFold2 models (Fig. 5B). Although AlphaFold2 currently provides a relatively precursive spatial structure, there were still mispredictions in the AflNmt protein structure, and similar imprecise examples in recent literature have found that the machine learning tool failed to predict protein fold switching or missense mutation effects (52, 53). There seems to be further room for AlphaFold2 improvement in the field of structure prediction.

## MATERIALS AND METHODS

### Strains and cultural conditions.

All experiments involving E. coli and WT and mutant A. flavus strains in this study are listed in Table S1 in the supplemental material. The fungal strains were cultured on GMM or XsMM medium for the growth study, and on YGT or YXsT medium for sclerotia formation and aflatoxin production analysis. The medium formulations used in this study are summarized in Table S2.

### Gene-editing strategy for generating mutant strains.

Primer information is provided in Table S3. To obtain the *Nmt^xylP^* strain, we used a homologous recombination approach to target the *Nmt* gene in A. flavus using a previously described method with slight modifications (26, 54). As shown in the flowchart (Fig. S1), four fragments (upstream of *Nmt*, screening marker-pyrG, xylose, and *Nmt*) were amplified with overlap extension PCR, and the PCR-amplified product was then transformed into the CA14 PTs protoplast cells. The positive transformants were verified by PCR at the genome level and by quantitative real-time PCR at the transcription level. The AflNmt-eGFP strain was constructed and identified by PCR using the same procedure.

### Phenotypic evaluation of strain.

For colony growth and sporulation analysis, GMM and XsMM medium plates were inoculated with 10^4^ spores and then cultured at 37°C in the dark for 4 days. The spore number was calculated following our previous method (54). For the spore germination study, the cultures (2 × 10^4^ spores/mL in GMM or XsMM fluid medium) were incubated in a shaker (180 r/min). Individual cell samples were collected after 6, 9, 12, and 16 h for microscopic observation. To evaluate sclerotium production, the WT and *Nmt^xylP^* strains (10^4^ spores) were cultured at 37°C in the dark for 14 days, the hyphae and spores were removed by 75% ethanol, and sclerotium growth was monitored (26). For aflatoxin detection, the two strains (10^5^ spores) were cultured in 10 mL of YGT or YXsT fluid nutrient medium at 29°C for 7 days. The aflatoxin extraction method was the same as a previous method (55); the toxin contents were then detected under a UV lamp.

### Fluorescent proteins localization.

The YES liquid cultures (AflNmt-GFP strain) were incubated on a shaker at 180 r/min for 10 h, and then mycelia were harvested and washed with phosphate-buffered saline (PBS) buffer. The mycelium samples were then stained with DAPI (excitation: 365 nm; emission: 420 nm) under a confocal laser microscope, and the AflNmt-GFP fluorescence signal was detected at a 488:507 excitation/emission wavelength ratio.

### Protein expression, purification and activity assays.

The cDNA fragment encoding AflNmt (residues 90 to 497) was subcloned into the pET-32a expression vector. The expression plasmid contains a 3C protease cleavage site between the Trx-6×His tag and the target gene. rAflNmt protein overexpression was carried out as described previously (56). The induced bacterial cells were harvested through centrifugation and disrupted by sonication. The soluble supernatant was collected and purified by Ni-NTA affinity chromatography. The purified Trx-6×His-rAflNmt protein was dialyzed against PBS buffer (PH 6.5) and further incubated with PreScission 3C protease at 4°C overnight. After centrifugation, the protein mixture was then purified by cation exchange and gel filtration chromatography.

rAflNmt activity was measured with a nonradioactive approach following a previously published protocol (57). In brief, the 85-μL mix reaction buffer [20 mM potassium phosphate, 0.1% Triton X-100, 2.7% dimethyl sulfoxide, 30 μM myristoyl-CoA solution, 6.3 nM rAflNmt protein, 8 μM 7-diethylamino-3-(4-maleimido-phenyl)-4-methylcoumarin] was first added to a 96-well black plate and incubated for 5 min at 25°C. A 15-μL volume of peptide substrate (H-Gly-Cys-Gly-Gly-Ser-Lys-Val-Lys-NH2) was added and the reaction was initiated, and then fluorescence intensity was recorded by excitation at 380 nm and emission at 470 nm.

### Protein crystallization and X-ray data.

The purified rAflNmt samples were concentrated for protein crystallization. Initial screening for rAflNmt was carried out using sitting-drop vapour diffusion based on commercial kits (JCSG Core I-IV Suite, NeXtal Biotechnologies, Montreal, Canada). The final crystallization optimization condition was 1 M HEPES (pH 6.5), 20% (wt/vol) PEG 6000.

The optimized crystals were directly frozen in liquid nitrogen with a 15% glycerol cryoprotectant. X-ray data collection experiments were performed at the Shanghai Synchrotron Radiation Facility (SSRF). Diffraction data were processed and scaled with the CCP4i2 program (58). The A. fumigatus Nmt structure (PDB: 4CAV) was selected as the molecular replacement model to solve the crystal phase problem using the Phenix phaser-MR programmer (59). Subsequent rAflNmt structure refinement involved multiple rounds of manual model building and adjustment with Coot (60). The final structure was refined with the Phenix-refine programm to R/Rfree values of 20.5/23.67 at 1.78 Å resolution. For visualization, the photographs of molecular structure and electron density map were generated with PyMol software (61). Details of the diffraction data collection and structure refinement statistics are given in Table 1.

**TABLE 1:** Data collection and refinement statistics^*a*^

Characteristic	AflNmt crystal (PDB: 8HBS)
Wavelength	0.979 Å
Resolution range	43.75−1.783 (1.847–1.783)
Space group	P 41
Unit cell dimensions	97.823, 97.823, 47.116, 90, 90, 90
No. of reflections	
Total	83,945 (7,343)
Unique	42,043 (3,384)
Multiplicity	2.0 (2.0)
Completeness (%)	96.39 (79.40)
Mean I/sigma(I)	22.73 (2.63)
Wilson B-factor	29.68
R-merge	0.0141 (0.248)
R-meas	0.01994 (0.3508)
R-pim	0.0141 (0.248)
CC1/2	0.999 (0.828)
CC*	1 (0.952)
Reflections used in refinement	41290 (3384)
Reflections used for R-free	1957 (164)
R-work	0.2050 (0.2888)
R-free	0.2367 (0.3622)
CC(work)	0.959 (0.762)
CC(free)	0.951 (0.711)
No. of non-hydrogen atoms	3,465
Macromolecules	3,210
Solvent	255
Protein residues	393
RMSD	
Bonds	0.007
Angles	1.07
Ramachandran	
Favored (%)	95.14
Allowed (%)	3.84
Outliers (%)	1.02
Rotamer outliers (%)	0.29
Clashscore	6.08
Avg B-factor	35.60
Macromolecules	35.12
Solvent	41.59

a Statistics are generated by PHENIX software (59). Statistics for the highest-resolution shell are shown in parentheses.
