# Supplementary material for: N-Myristoyltransferase, a Potential Antifungal Candidate Drug-Target for Aspergillus flavus
Source: Microbiol Spectr. 2022 Dec 21;11(1):e04212-22. doi: 10.1128/spectrum.04212-22 (PMC9927591; doi:10.1128/spectrum.04212-22)
Supplement: Supplemental file 1 — Supplemental material. Download spectrum.04212-22-s0001.pdf, PDF file, 0.8 MB [file spectrum.04212-22-s0001.pdf]

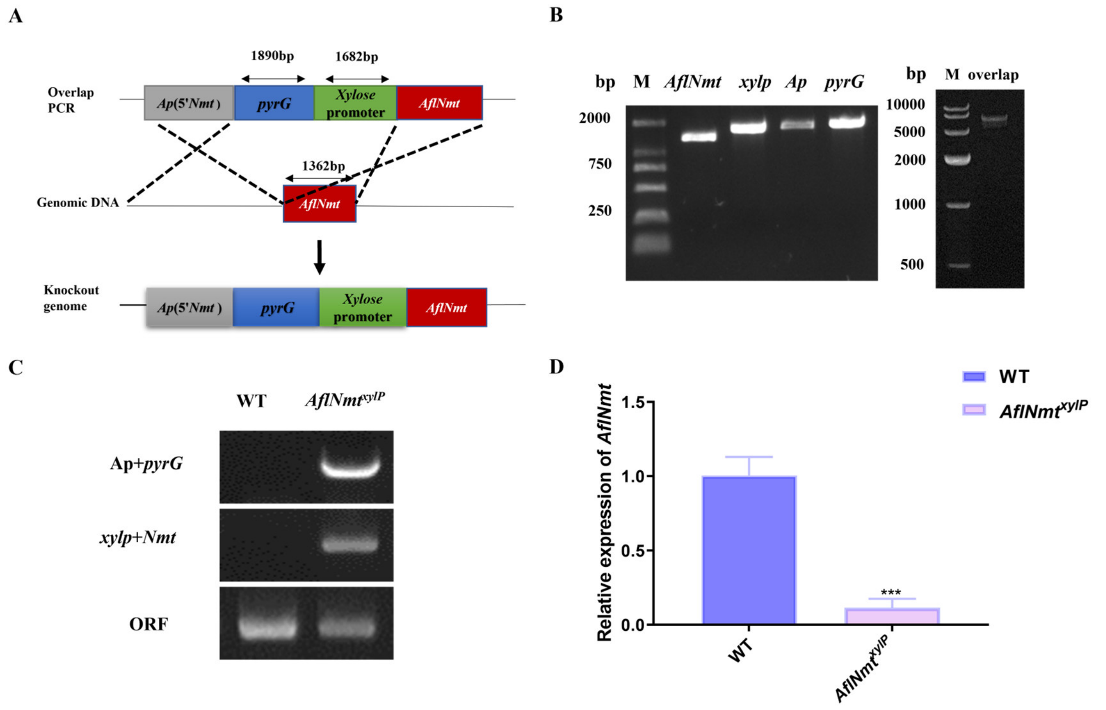

Figure S1. Construction of *AflNmt*^{xylP} strain. (A) Strategy and schematic diagram of the construction of *AflNmt*^{xylP} strain. The native promoter of *AflNmt* was replaced with the xylose-inducible promoter by using homologous recombination method in the *AflNmt*^{xylP} strain. Ap (5'*Nmt*): flanking regions upstream of *AflNmt gene*; *pyrG*: nutritional marker; *Xylose* promoter (*xylp*): xylose-inducible promoter. (B) Left panel: the PCR result of *AflNmnt*, *xylp*, Ap and *pyrG* fragments. *AflNmnt*: *AflNmnt gene* fragments; *xylp:* xylose-inducible promoter segment; Ap: flanking regions upstream of *AflNmt gene*; pyrG: nutritional marker segment. Right panel: these four fragments were fused by overlapping extension PCR; M: standard marker for molecular weight. (C) The homologous recombination transformant was verified by PCR. (D) The fluorescent quantitative analysis of Nmt expression levels in WT and *AflNmt*^{xylP} strains. *** means significant difference level ($P < 0.001$) based on t tests with three biological replicates.

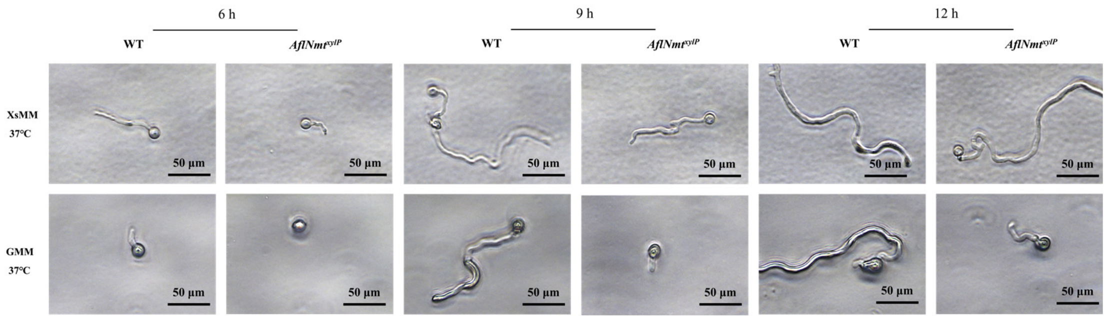

Figure S2 Microscopic observation of conidia germination for WT and $AflNmt^{xylP}$ strains. The results indicated that $AflNmt^{xylP}$ strains delayed germination at 37°C in GMM 和 XsMM liquid culture medium. The scale bar is 50 µm.

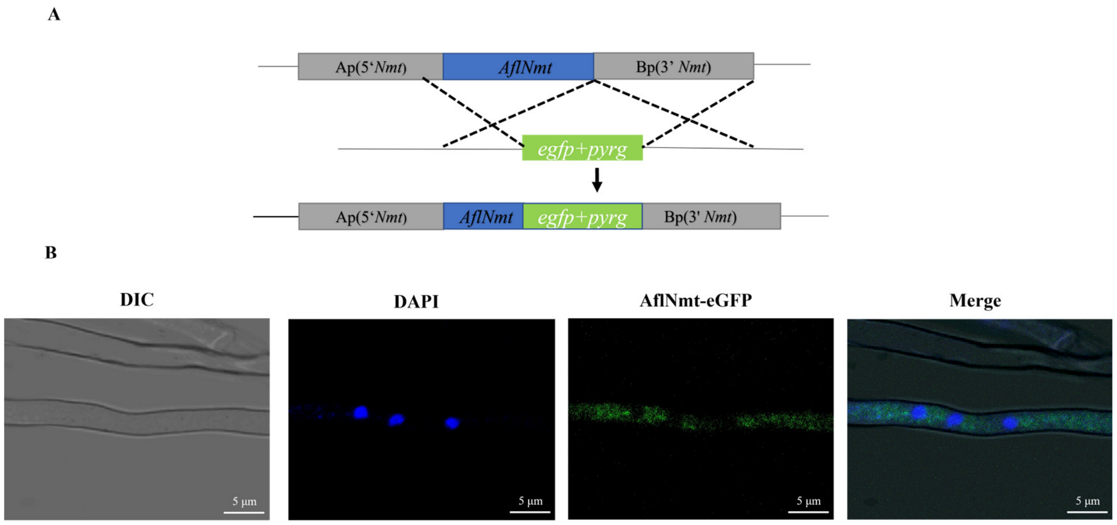

Figure S3 AflNmt is localized in the cytoplasm. (A) Strategy and schematic diagram of the construction of *AflNmt*-eGFP strain. Ap (5'*Nmt*): flanking regions upstream of *AflNmt gene*; egfp+pyrG: genome segment of enhanced green fluorescent protein and nutritional marker; Bp (5'*Nmt*): flanking regions downstream of *AflNmt gene*. (B) *AflNmt*-eGFP fusion protein was localized in the cytoplasm. The blue dye (DAPI) was used as a nucleus dye to locate nuclei. The scale bar is 5 µm.

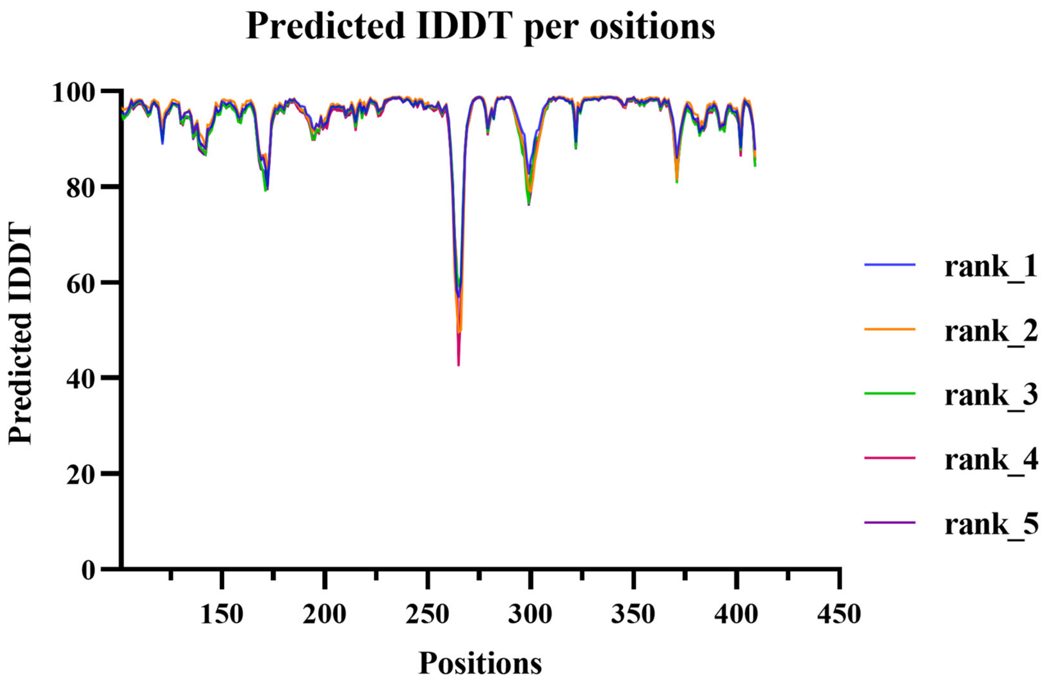

Figure S4. The value of Local Distance Difference Test (lDDT) for the five best prediction models was gived byAlphaFold2.

Table S1 Strains used in this study

Strain	Genotype description	Reference
A. flavus CA14 PTs	Δ*ku70*, Δ*pyrG*	(1)
wild type (WT)	Δ*ku70*, Δ*pyrG*::*pyrG*	this study
AflNmt^{xylP}	Δ*ku70*, Δ*pyrG*, Δ*AflNmt*::*pyrG*::*xyIP*::*AflNmt*	this study
AflNmt-eGFP	Δ*ku70*, Δ*pyrG*, Δ*AflNmt*::*pyrG*:: *AflNmt*::*eGFP*	this study
rAflNmt	*pET-32a-AflNmt*	this study

Table S2 Composition of culture media

Medium	Composition
XsMM	10 g/L xylose, 1 mol/L ammonium tartrate, 6 g/L $NaNO_3$, 0.52 g/L $MgSO_4$•$7H_2O$, KH_2PO_4 1.52 g/L, KCl 0.52 g/L,1 mL/L trace elements
YGT	5 g/L yeast extract, 20 g/L glucose, and 1 mL/L trace elements
YXsT	5 g/L yeast extract, 20 g/L xylose, and 1 mL/L trace elements
GMM	10 g/L glucose, 1 mol/L ammonium tartrate, 6 g/L $NaNO_3$, 0.52 g/L $MgSO_4$•$7H_2O$, KH_2PO_4 1.52 g/L, KCl 0.52 g/L, and 1mL/L trace elements
Trace element (100ml)	2.2 g $ZnSO_4$•7H2O, 1.1 g H_3BO_3, 0.5 g $MnCl_2$•$4H_2O$, 0.5 g $FeSO_4$•7H2O, 0.17 g $CoCl_2$•$5H_2O$, 0.16 g $CuSO_4$•5H2O, 0.005 g $(NH4)_6Mo_7O_{24}$•$5H_2O$, 4.45g Na_2EDTA

Table S3 Synthesized primers used in this study

Primers Name	Primers Sequence (5'-3')	Application
nmt-F1	TTGATGAAGCGGCTCGTGT	for *AflNmt*^{xylP} strain construction
nmt-R3	TGAAGAGCATTGTTTGAGGCGGGTTGA GTGCGGAAGGTTT	
xylP-F6	GCATCAGTGCCTCCTCTCAGACCTCGAG GTCGACGGAAGCG	
xylP-R7	GTTGGTTCTTCGAGTCGATGAATG	
nmt-F8	CATCGACTCGAAGAACCAACATGTCGGAC CCTAAGGATACCAA	
nmt-R10	AGGCATCGTTCATCAGGAGTAGA	

nmt-F2	GGTTCACCTCCCAGATACA	
nmt-R9	TCGTGGACGGCGTCAATAT	
pyrG-F4	GCCTCAAACAATGCTCTTCACCC	for *AflNmt-eGFP strain* construction
pyrG-R5	GTCTGAGAGGAGGCACTGATGC	
nmt-ORF-F	CAGACACAACCCGTACCCCGT	
nmt-ORF-R	TCACAACATAACAACGCCAAC	
nmt-eGFP-F1	GTCGGACCCTAAGGATACCAA	
nmt-eGFP-R3	GGCTCCAGCGCCTGCACCAGCTCCCAACAT AACAACGCCAACACC	
eGFP-F4	GGAGCTGGTGCAGGCGCTGGAGCCATGGTGA GCAAGGGCGAGGA	
eGFP-R5	GGGTGAAGAGCATTGTTTGAGGCTTACTTGTA CAGCTCGTCCATG	
nmt-eGFP -F6	GCATCAGTGCCTCCTCTCAGACCGTCAGTCGC CGAGCAAT	
nmt-eGFP -R8	CGCCCTTCGTTCAAGTCAG	
nmt-eGFP -F2	TGGTCTATCGGTCGGTGGG	
nmt-eGFP -R7	GGGAGTCGTGCCTGAACACTT	
AflsumO-c-qRT-F	CTTCTTTCTTGTTCTCAGCTG	for gene copy number identification
AflsumO-c-qRT-R	TAGGCGACCCCGTTCC	
AflsumO-g-qRT-F	ATCCTTTCCGTTCTCTGGC	
AfsumO-g-qRT-R	AGATTGTCAGCTCTGGTGC	
Aflnmt-c-qTR-F	CAGACACAACCCGTACCCCGT	
Aflnmt-c-qRT-R	TCACAACATAACAACGCCAAC	
Aflnmt-g-qRT-F	CGCAGGAAAGGAGCA	
Aflnmt-g-qRT-R	GACCGTTGATGGGAGG	
brlA-F	GCCTCCAGCGTCAACCTTC	for cDNA-qRT-PCR
brlA-R	TCTCTTCAAATGCTCTTGCCTC	
nsdC-F	GCCAGACTTGCCAATCAC	
nsdC-R	CATCCACCTTGCCCTTTA	
nsdD-F	GGACTTGCGGGTCGTGCTA	
nsdD-R	AGAACGCTGGGTCTGGTGC	
aflR-F	AAAGCACCCTGTCTTCCCTAAC	
aflR-R	GAAGAGGTGGGTCAGTGTTTGTAG	
aflS-F	CGAGTCGCTCAGGCGCTCAA	
aflS-R	GCTCAGACTGACCGCCGCTC	
Afnmt-OF	CAGACACAACCCGTACCCCGT	
Afnmt-OR	TCACAACATAACAACGCCAAC	
actin-F	ACGGTGTCGTCACAAACTGG	reference gene

actin-R	CGGTTGGACTTAGGGTTGATAG	
Lic-*Af*Nmt1-F	ACAAGGCCATGGCTGATATCGGATCCCTGGAA GTTCTGTTCCAGGGGCCCCAGACACAACCCGT [illegible]	for recombinant protein expression
Lic-*Af*Nmt1-R	AGTGGTGGTGGTGGTGGTGCTCGAGTCAC AACATAACAACGCCAAC	
Lic-*Hs*Nmt1-F	CCATGGCTGATATCGGATCCCTGGAAGTTCT GTTCCAGGGGCCCGAGGAGGCTAGCAAG	
Lic-*Hs*Nmt1-R	AGTGGTGGTGGTGGTGGTGCTCGAGTTATTG TAGCACCAGTCCAA	

Table.S4 Determination of the copy number of the *AflNmt* gene in *A. flavus*.

Sample	***AlfNmt***		***AflSumo***			
	**Ct valuve**	**CV(%)**	**Ct valuve**	**CV(%)**		
	**19.53±0.1**	**0.51**	**17.61±0.15**	**0.85**		
**WT**	**Initial Copy Number**				**Mean of CN**	**Round(CN)**
	AflNmt		***AflSumO***			
	**7029.212**		**7878.998**		**0.89**	**1**

Note: The tests were repeated with three replicates. Ct value is reported as mean ± SD, and CV denotes coefficient of variation. AflNmt and *AflSumO* stand for the initial copy number, CN represents copy number.

1. Chang P-K, Scharfenstein LL, Wei Q, Bhatnagar D. 2010. Development and refinement of a high-efficiency gene-targeting system for Aspergillus flavus. Journal of microbiological methods 81:240-246.
